# Supplementary material for: Flexible and Highly Sensitive Pressure Sensors Based on Microstructured Carbon Nanowalls Electrodes
Source: Nanomaterials (Basel). 2019 Apr 1;9(4):496. doi: 10.3390/nano9040496 (PMC6523954; doi:10.3390/nano9040496)
Supplement: Supplementary file 1 [file nanomaterials-09-00496-s001.pdf]

Supplementary Materials:

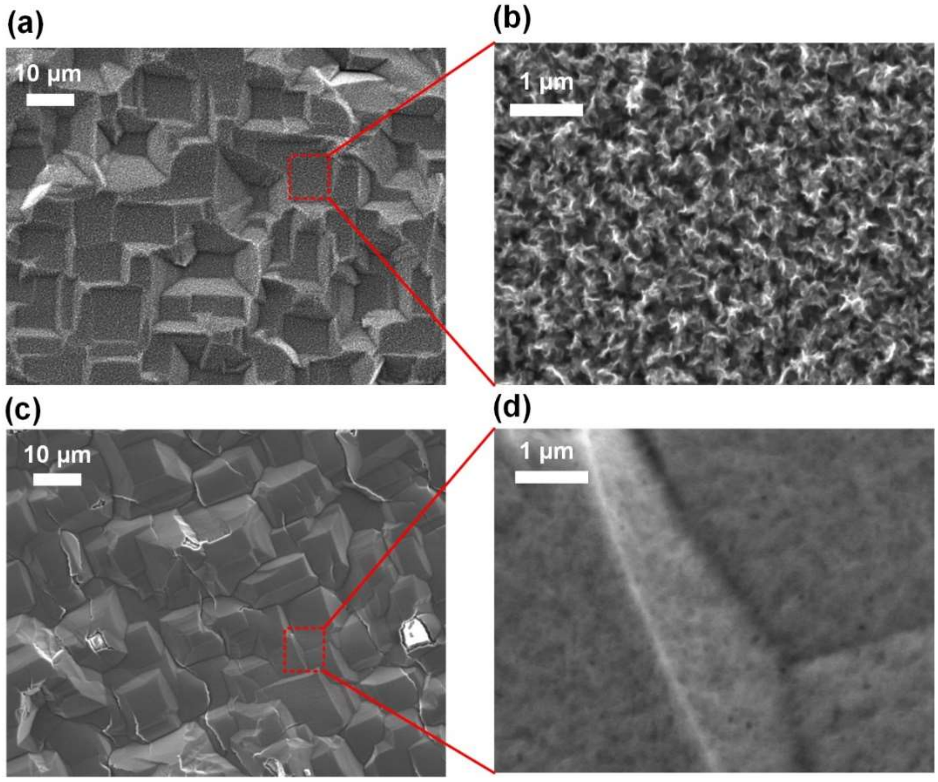

Figure S1. (a) SEM image of rough silicon wafer surface after carbon growing process. And (b) is the magnified image of the surface. The three-dimensional carbon structure can be clearly seen. (c) SEM image of the CNWs/PDMS electrode, (d) is the magnified image of the electrode. The interface between carbon and silicon wafer is relatively smooth

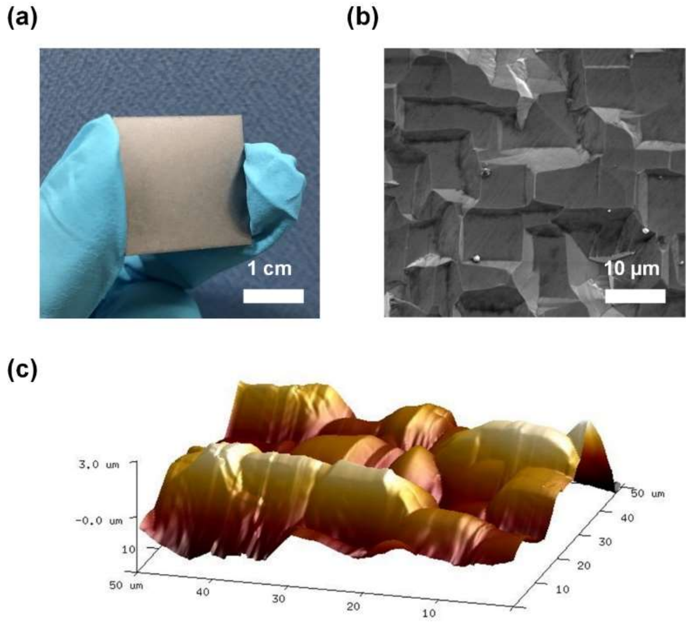

Figure S2. (a) Physical image of the rough silicon wafer. (b) SEM image of the rough silicon wafer. (c) Image of surface morphology of the CNWs/PDMS electrode detected by AFM.

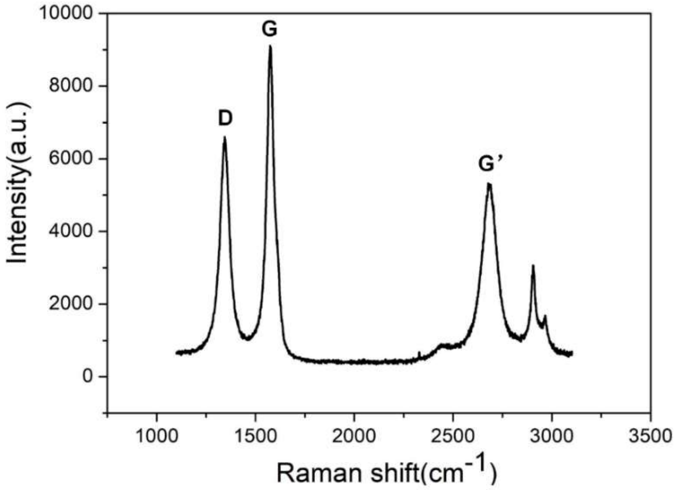

Figure S3. Raman spectra of carbon nano-walls measured by a 532nm laser.

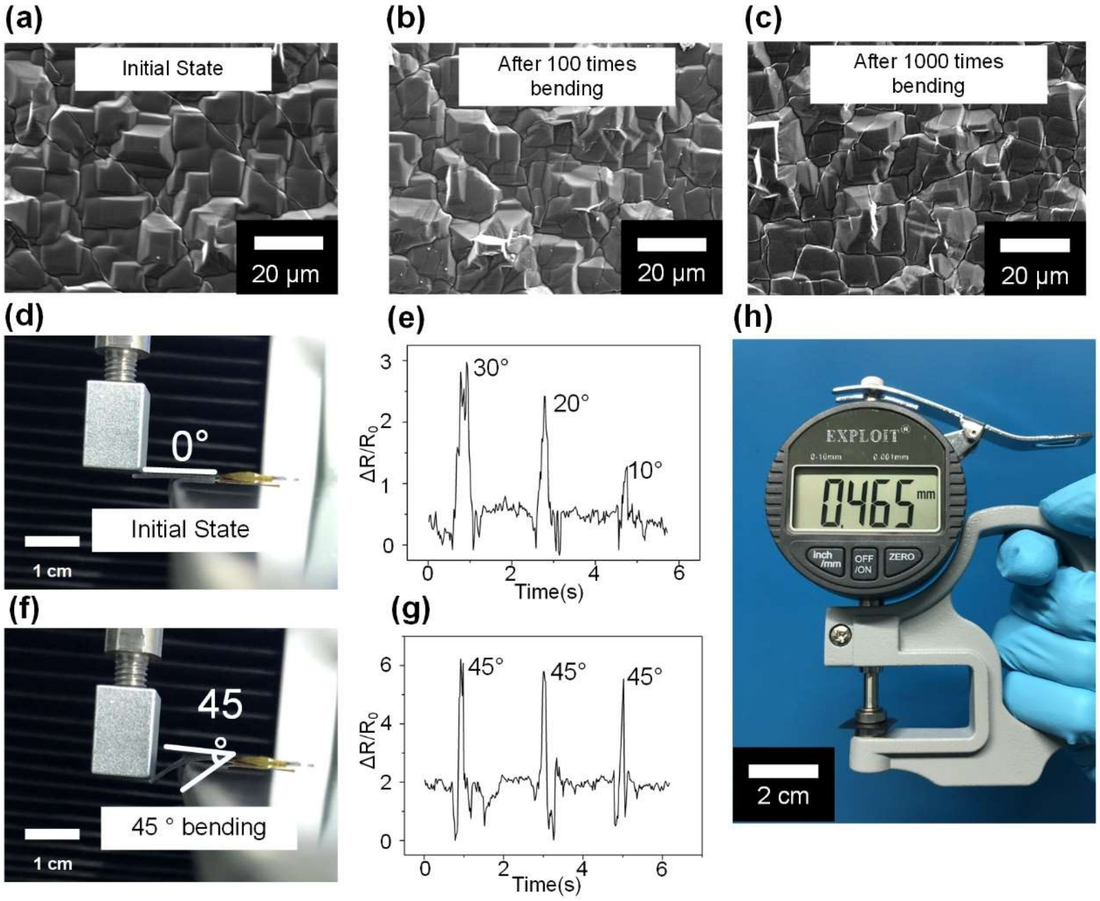

Figure S4. (a) Surface morphology of CNWs electrodes in the initial state; (b) Surface morphology after 100 cycles bending; (c) Surface morphology after 1000 cycles bending; (d) and (f) are physical pictures of electrodes at different bending angles; (e) Resistance changes at different bending angles; (g) Resistance changes under bending angle ~ 45 °; (h) Thickness testing of the CNWs electrode.

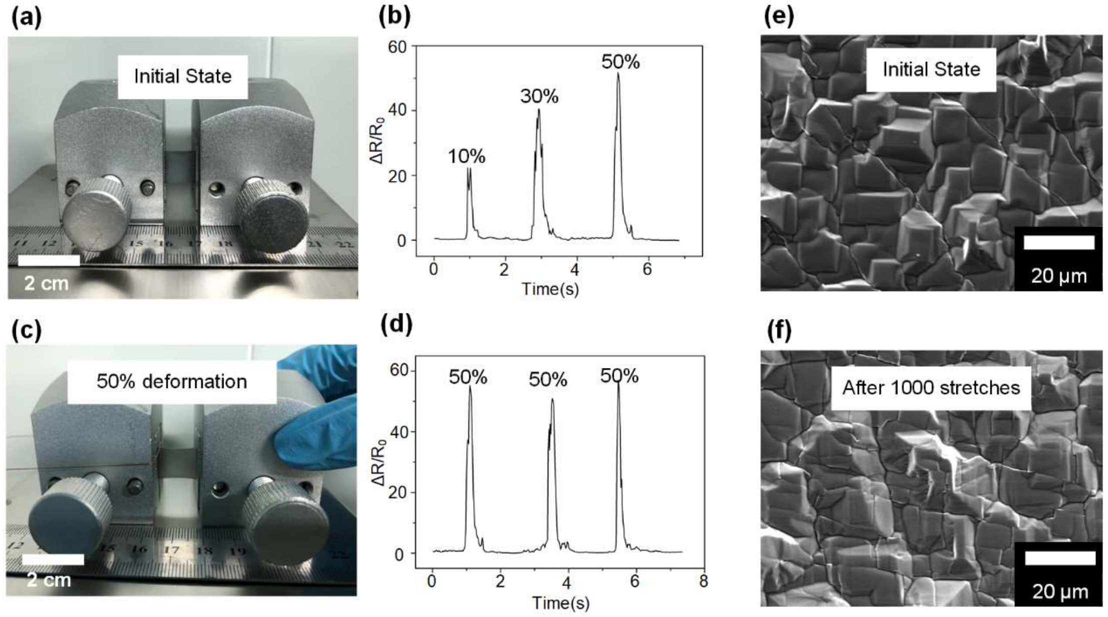

Figure S5. (a) The physical picture of the electrode tensile test in the initial state; (b) Resistance changes of the electrode under different tensile amounts; (c) Photograph of the CNWs electrode at 50% deformation; (d) Resistance changes of the electrode under 50% deformation; (e) Morphology of the electrode surface before the tensile test; (f) Surface morphology of the electrode after 1000 tensile tests.

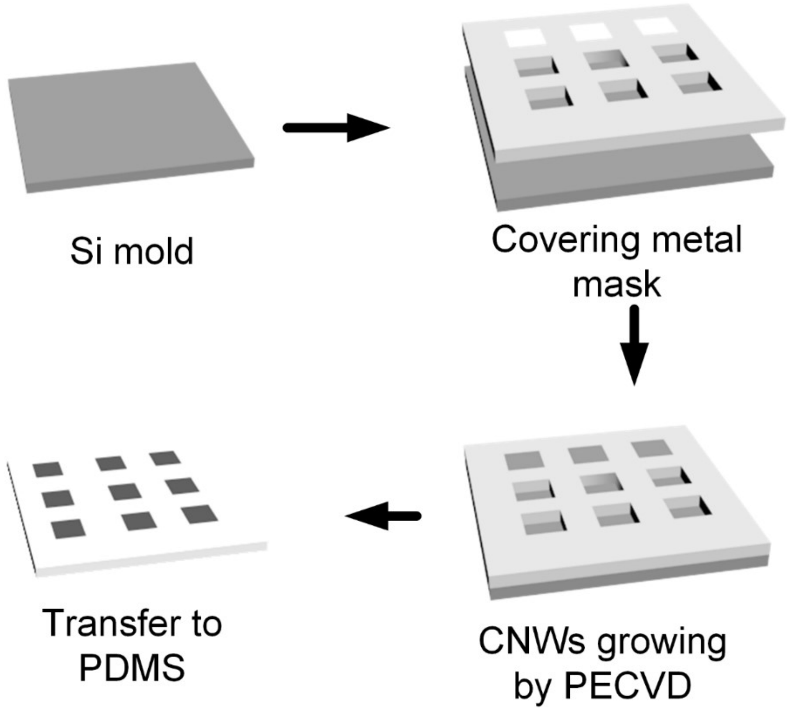

Figure S6. Schematic diagram of the assembly process of the sensor array.

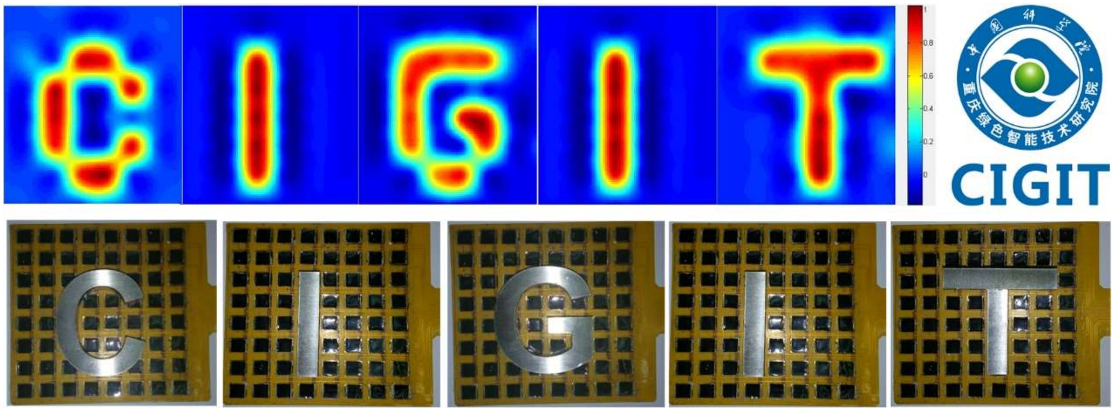

Figure S7. Metal stamps in the various shapes placed on the sensors array and corresponding mapping of the current changes.
